# Availability, Nutritional Profile and Processing Level of Food Products Sold in Vending Machines in a Spanish Public University

**DOI:** 10.3390/ijerph18136842

**Published:** 2021-06-25

**Authors:** Naiara Martinez-Perez, Marta Arroyo-Izaga

**Affiliations:** 1 Department of Nursing, Faculty of Medicine and Nursing, University of the Basque Country UPV/EHU, 48940 Leioa, Spain; naiara.martinez@ehu.eus; 2 Department of Pharmacy and Food Sciences, Faculty of Pharmacy, University of the Basque Country UPV/EHU, 01006 Vitoria-Gasteiz, Spain

**Keywords:** food environment, vending machines, snacks, drinks, nutrient profile model, university, public health

## Abstract

Background. Given the lack of data about the nutritional value and other determinants of the consumption of foods and drinks sold in vending machines in European universities and the relevance of this sector in Spain, it is necessary to obtain scientific data on this topic. The present study aimed to assess the availability, nutritional profile and processing level of food products from vending machines at a Spanish public university and to investigate differences in nutritional profile according to the cost and promotion. Methods. Cross-sectional descriptive study. Data from all products available (3894) were collected and analysed using the criteria of the Spanish Agency for Consumption, Food Safety and Nutrition and the United Kingdom nutrient profiling model. The items were also classified according to the degree of industrial processing through the NOVA system. Promotion was assessed, taking into account where products were displayed in vending machines. Results. The most common products were sweets (23.4% of the total options), coffee (20.3%) and salty snacks (11.7%). According to the combination of the two criteria used to assess nutritional profile, 48.6% of the products were classified as with low nutritional quality (LNQ). In addition, 73.8% of the items were categorised as “ultra-processed”. Foods (β = 0.31, 95% CI 0.24, 0.39, *p* < 0.001) and hot drinks (β = 0.46, 95% CI 0.39, 0.52, *p* < 0.001) with high nutritional quality (HNQ) were more likely to have higher prices than alternatives with LNQ. Both foods and cold drinks that support healthy dietary recommendations were promoted to a lesser extent than those with LNQ (*p* < 0.001). Conclusion. Almost half of the products were of LNQ and three-quarters had a high level of processing. Moreover, foods and cold drinks with LNQ were less expensive and more often promoted than alternatives with HNQ.

## 1. Introduction

Over the last decades, obesity rates have increased [1]. Precursors for obesity, such as poor nutrition and physical inactivity, that are related to physical and social environments are increasingly recognised [2]. Until recently, individuals were viewed as being solely responsible for their lifestyle choices with little regard given to the environments within which the choices were made. Both the social-ecological framework and the reciprocal determinism construct from social cognitive theory posit that environments and behaviours affect each other concurrently [3]. Environments that encourage choices that support healthy dietary recommendations could make it easier for individuals to achieve and/or maintain health goals, such as healthy weight status and an adequate micronutrient status, compared with environments that fail to support the choice of this type of food [4].

Studies of food environments to date have shown that many primary, secondary and post-secondary schools have an accessible and ubiquitous supply of foods and drinks that are not nutrient-dense and do not support healthy dietary recommendations [5,6,7,8]. Food and drink availability and accessibility constitute key elements for dietary behaviours, as they determine what can be eaten at a certain time and place [9]. Vending machines have been identified as sources of products with high energy value, high sugar and saturated fat, and low nutritional value [10,11,12]. Often, these products are the main source of food and drinks available in public spaces such as universities, where they are widespread across campuses and are particularly attractive to time-stressed, hungry students and workers of higher education institutions [7].

In vending machine audit studies from universities in Australia, the UK, and the USA, the proportions of items of low nutritional quality (LNQ) available ranged from 85% to 100% for foods and from 49% to 86% for drinks [6,7,13,14]. This fact is contrary to what is expected in the post-secondary education environment, which should promote healthy lifestyles that can set the stage for lifelong choices affecting health [15]. Tertiary institutions offer a convenient setting to target young adults in public health efforts, with 17.5 million students attending higher education institutions in 2018 in the European Union [16] and more than 40% of 30- to 34-year-olds having completed tertiary education [17].

Beyond taste and food preferences, other factors that may influence consumer purchasing from vending machines include pricing and promotion [18,19]. The price of vending machine products strongly influences consumer purchasing patterns, and when options with high nutritional quality (HNQ) are offered at a reduced cost, the sales of options with HNQ increase [10,20]. Consumers of products sold are also influenced by product logos, labels, and advertisement brand marketing [21,22]. To date, this last factor has been analysed through data related to any promotions on, inside or around the machine and the location of the machine [7,13].

Spain is one of the European leaders in vending machine use, with one machine for every 80 inhabitants [23] (the European average is of one for every 180 inhabitants [24]). In 2015, Spain dominated the European vending landscape along with five other countries: France, Germany, Italy, Netherlands and the UK [24]. Given the lack of data about the nutritional value and other determinants of the consumption of foods and drinks sold in vending machines in European universities [6,25] and the relevance of this sector in Spain, it is necessary to obtain scientific data on this topic. This work aimed to assess food products offered in vending machines at the University of the Basque Country (UPV/EHU) (in northern Spain), paying special attention to their nutritional profile and processing level, and to investigate differences in nutritional profile according to cost and promotion. This last factor (where items are displayed in the machine) is a new tool for the analysis of product promotion; it has not been previously used in vending machine assessment studies.

## 2. Materials and Methods

A cross-sectional study was carried out during the 2016/17 and 2017/18 academic years, specifically between February and November 2017. Vending machines of UPV/EHU in that period were managed by two contracted companies. During this period, the companies that were awarded the contracts did not change, and neither did the number of machines, nor the products offered, nor the price of the products. Data were recorded for all the vending machines of the three campuses of UPV/EHU, except for machines that, due to their location, are not usually accessible to undergraduate students (*n* = 24 machines) since they are in buildings earmarked for research.

### 2.1. Data Collection

Data related to the food and drinks offered at these points-of-sale were collected through a form developed for this study before data registration. This form included the following information: the number and type of machines and of food and drinks, building type (academic buildings, library, recreation facilities and other vending machines located in cafeterias or canteens, as described by other authors) [13], building floor, product description, location in the machine and portion price. The information collected regarding the product was as follows: flavour or ingredient variations, such as barbecue or plain potato chips, brand, portion weight, ingredients and nutrition labelling information (when it was available). The foods and drinks identified were assigned in one of the food/drink categories, according to the document on vending machines in schools developed by the Spanish Agency for Consumption, Food Safety and Nutrition (AECOSAN) and the Global Food Monitoring Group food categorisation system [26,27]. The price per portion was converted to euros for every 1 kg/L (€/kg or €/L) to make comparisons between products.

Cold and hot foods were grouped into the same category because the number of hot foods was small (0.9% of total foods). Duplicate items were also noted, as other authors previously did [5], to have complete information about the food offerings. In addition, digital photographs were taken of all machines, specifically a photograph of the entire machine, with an approximate distance of 2 m from the machine, and a photograph for every two rows, with an approximate distance of 1 m.

### 2.2. Food Product Assessment: Nutritional Profile and Processing Level

Information about food and drink nutritional composition was obtained from different sources, as follows (according to the order of preference): nutrition labelling, manufacturer’s website and/or the DIAL program 2.12, a food composition database developed for the Spanish population [28]. When the nutritional information required for this study was not available, these data were estimated from the ingredient list and the amount for each of them, using the DIAL program 2.12. The DIAL program was completed with the food composition tables of Mataix et al., (2009) whenever necessary [29]. For each product, the energy content and the following nutrients were estimated: proteins, sugars, dietary fibre, total fats, trans fatty acids (TFAs), saturated fatty acids (SFAs) and sodium content. These data were calculated per 100 g of product and per portion. In those products in which TFA data were not available in the nutrition labelling, nor on the manufacturer’s website or in the DIAL program, they were estimated using the report “Content of Trans Fatty Acids in Foods in Spain, 2015” (Agencia Española de Seguridad Alimentaria y Nutrición—AECOSAN) [30], and the food composition database of the USDA (United States Department of Agriculture) [31].

To indicate the nutritional quality of each food or drink item, the following nutrient profiling models (NPMs) were used: those proposed by the AECOSAN [26] and the United Kingdom (UK) NPM [29]. The former criteria are those designed for the food supply present in vending machines, canteens and kiosks in education centres. AECOSAN criteria have six components: energy, total fat, SFAs, TFAs, sugar and salt. These criteria sets the following limits per 100 g/mL of product: in foods ≤400 kcal, ≤15.6 g total fat, ≤4.4 g SFA, ≤1 g TFA, ≤30 g sugar and ≤1 g salt; and in drinks, ≤100 kcal, ≤3.9 g total fat, ≤1.1 g SFA, ≤0.25 g TFA, ≤7.5 g sugar and ≤0.25 g salt. Products that were over at least one of the cut-offs were considered of LNQ. These criteria focus on energy density and nutrients that have the potential to negatively impact on health or “at risk” nutrients, which can be a limitation when analysing the nutrient profiling. For this reason, we also used the United Kingdom NPM, which was developed by the UK Food Standards Agency [32]. This instrument is one of the most frequently validated models [33].

In addition to the “at risk” nutrients, the UK NPM also includes foods and nutrients considered to have a beneficial effect on health (i.e., fruit, vegetables, nuts, protein and fibre). The UK NPM uses a simple scoring system wherein points are allocated on the basis of the nutrient content of 100 g of food or drink. To do this, the nutrient content of each food and drink was assessed against a set of published criteria to determine whether it contains certain nutrients above or below particular thresholds. This model has seven components: energy, SFA, sugar, sodium, “fruit, vegetables and nuts”, fibre and protein; and provides a single score for any given food/drink product, based on calculating the number of points for “negative” nutrients that can be offset by points for “positive” nutrients. Points are awarded for energy, SFA, sugar and sodium (“A” nutrients) and fruit, vegetable and nut content, fibre and protein (“C” foods and nutrients). The amounts of these components were determined from the food/drink labelling (ingredient list, proportion of the ingredients listed on the label that have the highest percentages and nutrition labelling), manufacturer’s website and/or the dietary assessment that was carried out with the food composition database above-mentioned. The score for “C” foods and nutrients is subtracted from the “A” nutrients score to give a final score. If the score is <4 for foods or <1 for drinks, the product is classified as HNQ. When scores exceed these limits, however, the product is classified as LNQ.

Finally, the resulting categories after applying the two criteria mentioned above, AECOSAN and the UK Nutrient Profiling Model, were combined as follows: if a product had been classified as LNQ according to both classifications, it was considered LNQ. The rest of the products were categorised as NHQ. This criterion was agreed as being more rigorous than the one that would consider as LNQ those products that were classified as such according to one or both classification systems.

Additionally, the food or drink items were classified using the NOVA system [34], which categorises foods according to their nature, purpose and degree of industrial processing. This system distinguishes between the following groups: (i) unprocessed or minimally processed foods, (ii) processed culinary ingredients, (iii) processed foods and (iv) ultra-processed products. This last group, ultra-processed foods, are formulations made mostly or entirely from substances derived from foods (e.g., casein, lactose, whey, gluten, hydrogenated oils and maltodextrin, among others) and additives (e.g., colour stabilizers, flavour enhancers, non-sugar sweeteners and emulsifiers, among others), with little if any intact unprocessed or minimally processed. In the present study, the category “processed culinary ingredients” was not assessed because this type of product was not offered in the vending machines studied. However, these types of products were part of ready-to-eat foods such as salads with dressing that were classified as processed foods.

### 2.3. Location in the Vending Machine: Promotion of Products

The promotion of food and drinks was assessed through their location in the vending machines, based on the information recorded in the abovementioned photographs. It should be noted that none of the vending machines analysed had advertisements inside, around or on the vending machine fronts. Merchandising criteria were used to classify the products according to their location in the machine: ground level (at a height of less than 80 cm from the ground); hand level (from 80 cm to 120 cm); eye level (from 120 cm to 170 cm); and head level (more than 170 cm) [35]. To facilitate the data analysis, this classification was regrouped as follows: if the product was located at a height between 80 cm and 170 cm, it was considered to be promoted; in contrast, at heights above 170 cm or under 80 cm, the product was considered to be not promoted. Promotion was not evaluated for those products that were not directly visible to consumers (all hot drinks and some cold drinks), that is, those from vending machines that were either digital or non-transparent. In all these cases, the product selection panels, in which the list of products offered is displayed, were located at the level of the hands or eyes.

### 2.4. Quality Management

All data were collected by a single researcher (N.M.-P.) and reviewed by another researcher (M.A.-I.). We used unique vending machine identification numbers that were attached to each recording sheet. To check for quality data and derived indices (NPMs and level of processing), subsamples of machines and products were repeatedly examined. The data set was made available for analysis on a protected central data server. Access to the data is restricted to authorised members of the study.

### 2.5. Statistical Analysis

We hypothesised that more than half of the products offered are of LNQ, from a nutritional point of view, and have a high level of processing; foods with HNQ are more expensive, while foods with LNQ are less expensive and are displayed in places that favour their consumption. The data were analysed using SPSS for Windows (version 24.0, SPSS Inc., Chicago, IL, USA). The results are expressed as the mean (standard deviation, SD) and percentages. The distribution of values was examined for normality by the Kolmogorov-Smirnov-Lilliefors test. Non-parametric tests were performed for data that were non-normally distributed. The kappa coefficient was calculated to investigate the degree of agreement between the two NPMs (AECOSAN and UK NPM). The kappa results were interpreted as follows: values ≤0 no agreement, 0.1–0.20 none to slight, 0.21–0.40 fair, 0.41–0.60 moderate, 0.61–0.80 substantial and 0.81–1.00 almost perfect [36].

The chi-square test was used to compare the nutritional profiles, processing levels, product promotion and price, according to the type of food (solid food, cold drink and hot drink). The cost differences, taking into account the product’s nutritional profile, were determined by Mann-Whitney U test. Simple linear and binary logistic regression models were conducted to assess the associations of price (dependant variable, continuous) and promotion (dependant variable, categorical), respectively, with the nutritional profiles of products offered (independent variable resulting from the two criteria mentioned above, AECOSAN and the UK NPM, and the combination of both). Separate models were fit for each product category (hot and cold foods, cold drinks, hot drinks). All tests were two-sided, and *p*-values less than 0.05 were considered statistically significant.

## 3. Results

A total of 202 vending machines were studied across the three campuses (35 at the Álava/Araba campus, 102 at the Bizkaia campus, and 65 at the Gipuzkoa campus) of UPV/EHU. According to data provided by the Rector’s Office of the UPV/EHU, 42,598 students and 7482 workers were potential consumers of products available at the university’s vending machines; thus, there was one machine for every 248 members of the university community. Number of foods and drinks studied by category and subcategories are presented in Table 1.

Table 2 presents the number of vending machines according to campus and building type, the type of vending machine, and the number of products categorised by type. Overall, the percentage of drink machines (70.3%; cold drinks 37.6% and hot drinks 32.7%) was higher than those of only food and those with mixed offerings (*p* < 0.001) (Table 2), as was the percentage of drinks compared to that of food items (55.8% vs. 44.2%, *p* < 0.001; percentages calculated from the data presented in Table 1). Taking into account the building type, academic buildings had the largest number of vending machines.

A total of 3894 foods and drinks were surveyed in 40 buildings on three campuses. According to the NOVA system, most of the products offered were categorised as “ultra-processed”, specifically 73.8% of the total (99.7% of the cold and hot foods, 69.8% of the cold drinks and 35.3% of the hot drinks, *p* < 0.001) (Table 3). As shown in Table 4, the most common snack options were sweets (i.e., chocolate bars, biscuits) (23.4% of the total options), followed by coffee (20.3%) and salty snacks (11.7%). The most common product among cold and hot foods was sweet snacks (53% of the hot and cold foods), among cold drinks was bottled water (30.1% of the cold drinks), and among hot drinks was coffee (76% of the hot drinks). According to their nutritional profiles, more than half of the foods and drinks did not meet the AECOSAN criteria (53.9%) or the UK NPM criteria (51.5%).

The combination of the two criteria mentioned above, the AECOSAN and the UK NPM, showed that 48.6% of the products were classified as LNQ. It should be noted that among the non-carbonated drinks with added sugars, all met the AECOSAN criteria, while 94.7% did not meet the UK NMP criteria. These differences in the results obtained from the two NPMs are related to discrepancies in constructs and scoring criteria. These products had an average of 5.6 g of sugars per 100 mL (minimum 4.0 g and maximum 6.6 g). The sugar limit according to the AECOSAN criteria is ≤7.5 g in 100 mL drinks. Therefore, all these items met this and the rest of the AECOSAN criteria. Nevertheless, with the UK NMP, the scores for this “at risk” nutrient (sugar), as well as the one assigned to the foods and nutrients considered to have a beneficial effect on health, were low or null, so the results gave a high percentage of items classified as “with LNP”.

Comparison of the results obtained from the two NPMs showed an almost perfect agreement between UK NPM and AECOSAN (Supplementary Table S1). The lowest level of agreement was obtained for cold/hot foods; specifically, no agreement was observed for nuts (κ = 0.032) and sweet snacks (κ = 0.006), and the agreement was none to slight for cold sandwiches (κ = 0.112). The percentage of products that did not meet the AECOSAN and the UK NPM criteria was higher for hot and cold foods, followed by cold drinks and finally hot drinks. Among hot and cold foods, those products that met the AECOSAN and/or the UK NPM criteria to a lesser extent were salty snacks, hot sandwiches and sweets and chewing gum with added sugars; among cold drinks, dairy products; and among hot drinks, broths and hot chocolate. The AECOSAN criteria that were most frequently unfulfilled were the energy, total fat and SFA quantity in foods and the sugar content in drinks.

Regarding the comparison between the NPMs and processing level classification, in general a moderate agreement was observed between the NOVA system and each of the NPMs, separately and also combined (Supplementary Table S2). The lowest level of agreement between NPMs and NOVA system was obtained for cold/hot foods. Table 5 shows the relationships between nutritional profile and the promotion and price of products offered in vending machines. The hot and cold foods with LNQ, especially salty and sweet snacks, and hot drinks with LNQ were less expensive than the healthy foods (*p* < 0.001), whereas among cold drinks the results were the reverse (*p* < 0.05). Specifically, the price of bottled water (mean, 0.8; SD, 0.4 €/L) was significantly lower than that of soft drinks (mean, 2.5; SD, 1.1 €/L) (*p* < 0.001). Moreover, hot and cold foods with LNQ were promoted to a greater extent than healthy foods (*p* < 0.05). With respect to associations between product price and promotion, the promoted hot and cold foods were more expensive than those that were not promoted (mean, 18.6; SD, 11.6 €/kg vs. 12.1; 6.8 €/kg; *p* < 0.001), while among cold drinks, the reverse was true (mean, 1.8; SD, 1.2 €/L vs. 2.6; 1.4 €/L; *p* < 0.001).

Simple linear regression analyses showed that the product’s price was associated with its nutritional profile. Hot and cold foods (β = 0.31, 95% CI 0.24, 0.39, *p* < 0.001) and hot drinks that support healthy dietary recommendations (β = 0.46, 95% CI 0.39, 0.52, *p* < 0.001) were more likely to have higher prices than alternatives with LNQ (Supplementary Table S3). Among cold drinks, this association was inverse (β = −0.57, 95% CI −0.64, −0.50, *p* < 0.001). Moreover, both cold/hot foods and cold drinks that support healthy dietary recommendations were promoted to a lesser extent than those with LNQ (cold/hot foods, OR = 0.50, 95% CI 0.38, 0.65, *p* < 0.001; cold drinks, OR = 0.45, 95% CI 0.29, 0.70, *p* < 0.001) (Supplementary Table S4).

## 4. Discussion

The current study aimed to assess the nutritional profiles of foods and drinks sold in vending machines at a Spanish public university and to investigate differences in these profiles according to the cost and the product’s location in the machine. The most common products for sale in vending machines were sweets (i.e., chocolate bars, biscuits), coffee and salty snacks, results that agree with other authors, both at the university level [5,6] and in other settings [37,38]. Consistent with other studies conducted in university environments [5], it was found that almost half of the products offered for sale in vending machines at UPV/EHU were of LNQ (according to the combination of the two chosen criteria, the AECOSAN and the UK NPM criteria) and almost three-quarters had a high level of processing. Therefore, the initial hypothesis that more than half of the products offered are of LNQ and have a high level of processing was not completely confirmed. In any case, in the literature, the proportion of items sold with LNQ in vending machines available at campus universities was highly variable, probably because the criteria used were different [5,6,7,13]. It should also be noted that the processing level classification used in the present study showed a low level of agreement with the NPMs. This result is probably due to the fact that although ultra-processed foods usually are characterized by a high content of sugar, salt and/or fats, these contents do not always exceed the limits of the NPMs. In any case, ultra-processed foods had in general a worse nutrient profile than less-processed foods [39].

As other authors have pointed out, some of the potential reasons for filling vending machines with food with LNQ are the shelf life of the item and financial considerations [40]. Packaged snack products such as sweets and crisps often have a long shelf life and may not require refrigeration. However, an increasing number of vending companies are developing strategies to market vending machine products with HNQ that do not need to be refrigerated, and there are increasing numbers of refrigerated food-vending machines that can contain foods with HNQ that may be perishable [41,42]. Regarding financial considerations, marketing new products with HNQ may also be an effective way to promote sales and reduce any potential revenue loss [40,43,44].

In the present study, the analysis of nutritional profile by the type of product showed that those that met the recommendations the least were hot and cold foods (especially salty snacks, hot sandwiches and sweets and chewing gum with added sugars), followed by cold drinks (primarily dairy products) and hot drinks (broths and hot chocolate). Most foods, including salty snacks and hot sandwiches, were high in energy, total fat and SFAs, while drinks were high in sugar. These findings are in agreement with other studies examining the nutritional content of foods sold in vending machines in university [5,6], healthcare [37] and recreational [38] settings, confirming the poor nutritional quality of foods and drinks and the limited number of options that are lower in sugar, fat and saturated fat available from vending machines. Sufficient vending options that support healthy dietary recommendations, with health promotional messaging and with minimal processing should be guaranteed to increase the purchases of products with HNQ from vending machines [12].

With respect to the level of agreement between the two NPMs used, it should be noted that it was almost perfect for total products and for cold and hot drinks; however, the lowest level of agreement was obtained for cold/hot foods. These differences in the results obtained from the two NPMs, in the present study, could be related to discrepancies in constructs and scoring criteria for the models used. In fact, UK NPM penalizes high content in sodium, SFA and sugar, but the scores obtained for these products are offset by the positive points associated with the components “fruit, vegetables and nuts” and fibre; a fact that does not happen in the case of the AECOSAN model. Therefore, the percentage of products, in particular, nuts, sweet snacks and cold sandwiches, classified as “with LNP” was higher with AECOSAN’s criteria than with UK NMP.

Regarding the differences in nutritional profile according to cost, hot and cold foods and hot drinks with LNQ were consistently less expensive than alternatives with HNQ, while among cold drinks, the results were the reverse (encouragingly, water was often less expensive than soft drinks). The association found between food and drinks with LNQ and high price agrees with the findings of other authors [7,45]. However, not all studies found this association. For example, Ng et al., (2019) did not find statistically significant price differences and argued that this finding might be due to the few options of HNQ available [46]. Various authors have pointed out that the price effects of vending machine products were strong on consumer purchasing patterns [10,19], and when options of HNQ were offered at a reduced cost, the sales of options with HNQ increased [20,47,48]. However, the effects of price and product interventions on profits are still inconclusive [49,50]. Concern that price changes would reduce sales and profit may impede managers from offering food and drink choices with HNQ. Therefore, additional studies based on longitudinal data are needed to develop an evidence base concerning the potential effectiveness of pricing interventions among this university community to help improve food consumption patterns.

In addition, products with LNQ were more often promoted, as they were mostly located at the eye and hand levels. These results agree with previous research that showed that food marketing promotes mainly low-nutrition foods and drinks [51]. The layouts and specific product placements that maximise purchases of particular foods in supermarkets have been widely demonstrated to be effective in manipulating buying behaviour [52]. Given the results obtained in this study and data from the literature, we consider it necessary to implement strategies that combine sufficient, price-reduced vending options with HNQ located in strategic spaces to increase purchases of products with HNQ from vending machines. To ensure the implementation of these changes on campus, policies should be implemented, including policies at the European, state and institutional levels.

At the European and state levels, council and governmental regulations could require that all food and drink products offered in higher education institutions meet healthy nutritional criteria, according to current national and international evidence-based nutritional recommendations. Government regulations could guarantee the nutritional quality of the food and drinks offered in universities. At the institutional level, stakeholders, such as decision-makers (e.g., vice management of contracting), food service companies and the university community (students and workers), must be involved in encouraging changes in this regard [5]. After this study, the UPV/EHU implemented some measures to improve the nutritional quality and sustainability of the food and drink products from vending machines [53] through the bid specifications of contracts related to food services. However, difficulties in actually meeting the guidelines and compliance monitoring have been noted since specific plans for monitoring implementation were not provided.

To date, health policy has been developed to improve the availability of food and drinks with HNQ for health facilities [54] and primary and secondary schools [55,56,57], among others. Currently, in Spain, specific legislation applies to schools [58]. Furthermore, in 2018, the Basque government started a pilot project to increase the number of foods with HNQ in vending machines. This Basque government project set the objective that 50% of the products offered in schools, hospitals, universities and also in companies should be of HNQ [59]. Additional research is needed to determine whether the presence of items with HNQ in vending machines makes a difference in consumption behaviours when up to 50% of vending machine items remain of LNQ. In terms of setting vending nutrition/snack policies, higher education institutions/employers have been less proactive, and there are no guidelines specifically developed for university settings. Therefore, it is necessary to develop clear policy recommendations specifically for tertiary education settings to guide university administrators to become aware of the issues and to demand change from their vending suppliers. Given the lack of policies of this type in Spain and Europe, those developed and implemented in other countries, such as the United States, could be taken as an example [60,61].

To better contextualise the findings of this research, some limitations need to be acknowledged. First, as data on food and drinks were registered at one point in time, changes in the food supply were not taken into account. However, these changes are usually few in number during the valid period of the supply contract. Second, the sales or consumption of products from vending machines was not assessed; to overcome this limitation, we plan to analyse these data shortly. Despite these limitations, there are several strengths associated with this study. First, to our knowledge, few studies have focused on the nutritional profiles of vending foods and drinks at Spanish universities [25], and none have analysed differences in the nutritional profiles according to price and where products were displayed in vending machines. Second, this research included an analysis of all the vending machines accessible to students and workers of a Spanish public university. Other similar studies assessed only some of the university campuses [5,6,45], which decreases the external validity of the findings. Third, we used a fairly new tool for vending machine assessment studies, digital photography [13,21]. This method is highly accurate, reliable and time effective and allows data acquisition for uninterrupted evaluation of the food environment [13,21]. Finally, we applied a tool for the analysis of product promotion that has not been previously used in vending machine assessment studies. This merchandising criterion based on the location of items in the machine could be useful to promote foods and drinks with HNQ.

## 5. Conclusions

Our findings suggest that almost half of the products from vending machines at UPV/EHU were of LNQ, from a nutritional point of view, and almost three-quarters had high levels of processing. Therefore, access to food options with HNQ is limited. Moreover, foods and cold drinks with LNQ were less expensive and more often promoted (i.e., were mostly located at hand or eye level) than alternatives with HNQ. These findings can be useful for developing interventions and policies targeted at improving the healthiness of products from vending machines on campuses, and these environmental changes could make choices with HNQ possible and easier. Future research should focus on the design, implementation and evaluation of intervention strategies and the effect on profits.

## Figures and Tables

**Table 1:** Numbers of food and drink products by category and subcategory.

Product	Category	Subcategory	*n* (%)
Cold/hot foods	Sweet snacks	Bakery and pastry products	207 (12.0)
		Biscuits	191 (11.1)
		Cereal bars	96 (5.6)
		Chocolate	78 (4.5)
		Chocolate bars	293 (17.0)
		Jellybeans	48 (2.8)
	Salty snacks	Bakery products (for example, breadsticks)	104 (6.0)
		Other bakery products (for example, empanadillas)	2 (0.1)
		Chips	109 (6.3)
		Crackers	14 (0.8)
		Fried corn	27 (1.6)
		Extruded snacks (for example, “Doritos”)	195 (11.3)
		Rice/corn cakes	5 (0.3)
	Salads	Salads	1 (0.1)
	Sandwiches	Cold sandwiches	168 (9.8)
		Hot sandwiches	16 (0.9)
	Nuts	Fried nuts with salt	68 (3.9)
		Natural or toasted nuts without salt	1 (0.1)
		Natural or toasted nuts with salt	10 (0.6)
	Fresh fruit	Fresh fruit	2 (0.1)
	Other food	Candies with added sugars	5 (0.3)
		Candies with sweeteners	1 (0.1)
		Chewing gum with sweeteners	82 (4.8)
Total			1723
Cold drinks	Bottled water	Bottled water	341 (30.1)
	Carbonated drinks	Carbonated drinks with sugar and sweeteners	6 (0.5)
		Carbonated drinks with sweeteners	148 (13.1)
		Sugar sweetened carbonated drinks	232 (20.5)
		Carbonated drinks with juice	39 (3.5)
		Soda	1 (0.1)
	Non-carbonated drinks	Isotonic drinks	111 (9.8)
		Non-carbonated drinks with sugar and sweeteners	28 (2.5)
		Soft drinks with juice	56 (4.9)
		Sugar sweetened non-carbonated drinks	1 (0.1)
	Beer	Alcohol-free beer	1 (0.1)
		Beer with alcohol	2 (0.2)
	Dairy cold drinks	Coffee	5 (0.4)
		Chocolate	5 (0.4)
	Fruit juice	Concentrated juices	1 (0.1)
		Juice with milk	120 (10.6)
		Nectars	36 (3.2)
Total			1133
Hot drinks	Milk	Milk	64 (6.2)
	Coffee	Small coffee	72 (6.9)
		Large coffee	73 (7.0)
		Coffee with a little milk	73 (7.0)
		Coffee with milk	74 (7.1)
		Bonbon coffee	1 (0.1)
		Irish coffee	1 (0.1)
		Hazelnut coffee	29 (2.8)
		Cappuccino	73 (7.0)
		Hazelnut cappuccino	8 (0.8)
		Italian cappuccino	31 (3.0)
		Mocha	32 (3.1)
		Milk with a little coffee	55 (5.3)
		Small decaffeinated coffee	64 (6.1)
		Large decaffeinated coffee	28 (2.7)
		Decaffeinated coffee with a little milk	66 (6.4)
		Decaffeinated coffee with milk	66 (6.4)
		Decaffeinated cappuccino	29 (2.8)
		Decaffeinated hazelnut cappuccino	3 (0.3)
		Milk with a little decaffeinated coffee	11 (1.1)
	Hot chocolate	Hot chocolate	68 (6.5)
		Hot chocolate with milk	27 (2.6)
		Viennese chocolate	31 (3.0)
		White chocolate	1 (0.1)
	Infusions	Tea	2 (0.2)
		Tea with lemon	46 (4.4)
	Other hot drinks	Hot water	1 (0.1)
		Broths	9 (0.9)
Total			1038

**Table 2:** Types of vending machines and products sold at the University of the Basque Country (Spain) by campus.

Campus	Building Types	Type of Food/Drink Vending Machine					Type of Product					
		Total, *n* (%)	HF	CD	HD	M	Total, *n* (%)	HF	CD	HD	CF	*p* ^a^
			%					%				
A	Academic buildings	24 (68.6)	-	61.5	72.7	72.7	537 (70.8)	-	64.8	73.9	73.1	
	Library	7 (20.0)	-	23.1	18.2	18.2	157 (20.7)	-	21.5	18.5	21.3	
	Other ^b^	4 (11.4)	-	15.4	9.1	9.1	65 (8.6)	-	13.7	7.6	5.6	
	Total	35 (100.0)	-	37.1	31.4	31.4	759	-	30.7	24.2	45.1	**0.010**
B	Academic buildings	94 (92.2)	100.0	87.8	93.8	96.2	1756 (91.1)	100.0	82.5	93.5	96.3	
	Library	1 (1.0)	-	-	3.1	-	16 (0.8)	-	-	3.3	-	
	Recreation facilities	2 (2.0)	-	2.4	-	3.8	44 (2.3)	-	2.5	-	3.8	
	Other ^b^	5 (4.9)	-	9.8	3.1	-	112 (5.8)	-	15.3	3.3	-	
	Total	102 (100.0)	2.9	39.8	25.2	31.1	1928	0.4	32.6	25.5	41.5	**0.004**
G	Academic buildings	49 (75.1)	-	72.7	82.6	70.0	919 (76.1)	-	75.7	82.3	72.4	
	Library	5 (7.7)	-	4.5	8.7	10.0	104 (8.6)	-	5.5	8.8	9.9	
	Other ^b^	11 (16.9)	-	22.7	8.7	20.0	184 (15.2)	-	18.8	8.8	17.6	
	Total	65 (100.0)	-	30.8	30.8	38.5	1207	-	22.5	30.0	47.5	**<0.001**
Total	Academic buildings	167 (82.7)	100.0	78.9	86.4	82.5	3212 (82.5)	100.0	77.2	86.1	83.7	
	Library	13 (6.4)	-	5.3	7.6	7.0	277 (7.1)	-	5.7	7.9	7.6	
	Recreation facilities	2 (1.0)	-	1.3	-	1.8	44 (1.1)	-	1.2	-	1.7	
	Other ^b^	20 (9.9)	-	14.5	6.1	8.8	361 (9.3)	-	15.8	6.0	7.0	
	Total	202 (100.0)	1.5	37.6	32.7	28.2	3894	0.2	29.1	26.7	44.0	**<0.001**

Abbreviations: HF, hot foods; CD, cold drinks; M, mixed hot foods/drink; HD, hot drinks; CF, cold foods; A, Araba/Álava; B, Bizkaia; G, Gipuzkoa. Note: ^a^ Chi-square was used to assess differences in the frequency distribution of types of products by Campus. Significant *p*-values are highlighted in bold; ^b^ Other: vending machines in cafeterias or canteens in several buildings.

**Table 3:** Processing levels of products offered in food and drink vending machines at the University of the Basque Country (Spain).

Type of Product (*n*)	NOVA Classification System ^a^, %		
	Not Processed ^b^	Processed	Ultra-Processed
Cold/hot foods			
Fresh fruit (2)	100	-	-
Nuts (79)	1.3	1.3	97.5
Salty snacks (456)	-	-	100.0
Salads (1)	-	100.0	
Sandwiches			
cold sandwiches (168)	-	-	100.0
hot sandwiches (16)	-	-	100.0
Sweets and chewing gum			
with added sugars (5)	-	-	100.0
with sweeteners (83)	-	-	100.0
Sweet snacks (913)	-	-	100.0
Total (1723)	0.2	0.1	99.7
Cold drinks			
Beer (3)	-	-	100.0
Bottled water (341)	100	-	-
Carbonated drinks			
without sugar or sweeteners (1)		100.0	
with added sugars (234)	-	-	100.0
with sweeteners (148)	-		100.0
with added sugars and sweeteners (43)	-	-	100.0
Dairy products (10)	-	-	100.0
Fruit juice (157)	-	-	100.0
Non-carbonated drinks			
with added sugars (19)	-	-	100.0
with added sugar and sweeteners (177)	-	-	100.0
Total (1133)	30.1	0.1	69.8
Hot drinks			
Broths (9)	-	-	100.0
Coffee (789)	71.0	-	29.0
Hot chocolate (127)	-	-	100.0
Hot water (1)	100.0	-	-
Infusions (48)	100.0	-	-
Milk (64)	100.0	-	-
Total (1038)	64.7	-	35.3
Total (3894)	26.1	0.1	73.8

Note: ^a^ Monteiro et al., 2016; ^b^ Not processed or minimally processed.

**Table 4:** Nutritional profiles of products offered in vending machines at the University of the Basque Country (Spain).

		Percentage Not Meeting Criteria									
Type of Product	*n* (%)	AECOSAN Criteria ^a,b^, %							UK NPM Criteria ^b,c^, %	*p* ^d^	AECOSAN + UK NPM ^a,c^, %
		Energy	Total Fat	SFA	TFA	Sugars	Salt	Total			
Cold/hot foods											
Fresh fruit	2 (0.1)	-	-	-	-	-	-	-	-	-	-
Nuts	79 (4.6)	96.2	-	1.3	55.7	1.3	88.6	98.7	55.7	0.443	55.7
Salty snacks	456 (26.5)	97.4	77.6	48.2	87.7	1.3	92.3	100.0	87.7	-	87.7
Salads	1 (0.1)	-	-	-	-	-	-	-	-	-	-
Cold sandwiches	168 (9.8)	2.4	13.1	3.0	52.0	-	94.6	94.6	50.6	**0.002**	52.0
Hot sandwiches	16 (0.9)	50.0	62.5	50.0	72.7	-	81.3	100.0	62.5	-	72.7
Sweets and chewing gum											
with added sugars	5 (0.3)	40.0	-	-	100.0	100.0	-	100.0	100.0	-	100.0
with sweeteners	83 (4.8)	-	-	-	-	-	-	-	-	-	-
Sweet snacks	913 (53.0)	88.5	88.9	91.2	93.8	75.0	8.7	97.8	95.8	0.577	93.8
Total ^e^	1723	77.9	69.5	61.9	81.4	40.5	43.1	93.3	82.5	**<0.001**	81.4
Cold drinks											
Beer	3 (0.3)	-	-	-	-	-	-	-	33.3	-	-
Bottled water	341 (30.1)	-	-	-	-	-	-	-	-	-	-
Carbonated drinks											
without sugar or sweeteners	1 (0.2)	-	-	-	-	-	-	-	-	-	-
with added sugars	234 (20.6)	-	-	-	89.7	89.7	-	89.7	89.7	-	89.7
with sweeteners	148 (13.1)	-	-	-	-	-	-	-	-	-	-
with added sugar and sweeteners	43 (3.8)	-	-	-	86.0	86.0	-	86.0	88.4	**<0.001**	86.0
Dairy products	10 (0.8)	-	-	-	100.0	100.0	-	100.0	100.0	-	100.0
Fruit juice	157 (13.9)	-	-	-	2.5	3.2	-	3.2	9.6	**<0.001**	2.5
Non-carbonated drinks											
with added sugars	19 (1.7)	-	-	-	-	-	-	-	94.7	-	-
with added sugar and sweeteners	177 (15.6)	-	-	-	47.5	47.5	-	47.5	80.2	**<0.001**	47.5
Total ^e^	1133	-	-	-	30.5	30.5	-	30.5	38.5	**<0.001**	30.5
Hot drinks											
Broths	9 (0.9)	-	-	-	100.0	-	100.0	100.0	100.0	-	100.0
Coffee	789 (76.0)	-	-	-	1.3	1.3	-	1.8	2.1	**<0.001**	1.3
Hot chocolate	127 (12.2)	-	-	-	100.0	100.0	-	100.0	100.0	-	100.0
Hot water	1 (0.1)	-	-	-	-	-	-	-	-	-	-
Infusions	48 (4.6)	-	-	-	-	-	-	-	2.1	-	-
Milk	64 (6.2)	-	-	-	-	-	-	-	-	-	-
Total ^e^	1038	-	-	-	14.1	13.2	0.9	14.1	14.5	**<0.001**	14.1
Total	3894	34.5	30.8	27.4	48.6	30.3	19.3	53.9	51.5	**<0.001**	48.6

Abbreviations: NPM, nutrient profiling model; SFA, saturated fat acids; TFA, *trans* fatty acids. Note: ^a^ AECOSAN, 2010; ^b^ The same product may not meet more than one criterion, and therefore, the sum of the criteria does not result in the total percentage of products that do not fulfil AECOSAN criteria; ^c^ Department of Health of the UK, 2011; ^d^ Chi-square was used to assess differences between percentage not meeting the AECOSAN criteria and the UK NPM criteria. Significant *p*-values are highlighted in bold; and ^e^ the total rows present percentages with respect to the total of each type of product.

**Table 5:** Relationships between nutritional profile and the promotion and price of products offered in vending machines at the University of the Basque Country (Spain).

Type of Product (*n*)	Price, €/kg or L			*p* ^c^	Promoted Products ^a^			*p* ^c^
	Total, Mean (SD)	PHNQ (*n* = 1659), Mean (SD)	PLNQ ^b^ (*n* = 1894), Mean (SD)		Total ^d^, *n*	PHNQ (*n* = 27), %	PLNQ (*n* = 184), %	
Cold/hot foods								
Fresh fruit (2)	3.8 (0.2)	3.8 (0.2)	-	-	-	-	-	-
Nuts (79)	12.6 (4.5)	12.5 (6.4)	12.7 (2.3)	0.513	69	28 (40.6)	41 (59.4)	0.099
Salty snacks (456)	14.3 (6.5)	22.0 (6.1)	13.3 (5.9)	**<0.001**	356	32 (9.0)	324 (91.0)	**<0.001**
Salads (1)	6.9 (-)	6.9 (-)	-	-	-	-	-	-
Cold sandwiches (168)	7.3 (2.0)	6.7 (2.1)	10.3 (2.5)	**0.003**	36	18 (50.0)	18 (50.0)	0.936
Hot sandwiches (16)	9.6 (2.8)	7.6 (2.3)	7.0 (1.6)	0.057	8	-	8 (100.0)	0.200
Sweets and chewing gum								
with added sugars (5)	26.3 (0.0)	-	26.3 (0.0)	-	3	-	3 (100.0)	-
with sweeteners (83)	43.2 (12.7)	43.2 (12.7)	-	-	83	83 (100.0)	-	-
Sweet snacks (913)	18.6 (10.2)	32.8 (10.5)	17.7 (9.5)	**<0.001**	797	56 (7.0)	741 (93.0)	**0.010**
Total (1723)	17.2 (11.1)	24.3 (16.6)	15.6 (8.6)	**<0.001**	1352	217 (16.0)	1135 (84.0)	**<0.001**
Cold drinks								
Beer (3)	3.3 (0.4)	3.3 (0.4)	-	-	-	-	-	-
Bottled water (341)	0.8 (0.4)	0.8 (0.4)	-	-	58	58 (100.0)	-	-
Carbonated drinks								
without sugars or sweeteners (1)	3.0 (-)	3.0 (-)	-	-	1	1 (100.0)	-	-
with added sugars (234)	2.6 (1.5)	1.3 (0.6)	2.8 (1.6)	**<0.001**	35	-	35 (100.0)	**<0.001**
with sweeteners (148)	2.0 (0.5)	2.0 (0.5)	-	-	21	21 (100.0)	-	-
with added sugar and sweeteners (43)	2.4 (0.7)	3.0 (0.5)	2.3 (0.7)	0.042	5	2 (40.0)	3 (60.0)	**1.000**
Dairy products (10)	6.0 (0.4)	-	6.0 (0.4)	-	1	-	1 (100.0)	-
Fruit juice (157)	2.5 (0.6)	2.5 (0.6)	3.5 (1.1)	**0.041**	8	6 (75.0)	2 (25.0)	**0.013**
Non-carbonated drinks								
with added sugars (19)	1.9 (1.0)	1.9 (1.0)	-	-	9	9 (100.0)	-	-
with added sugar and sweeteners (177)	2.5 (0.7)	2.7 (0.8)	2.3 (0.5)	**0.004**	21	13 (61.9)	8 (38.1)	0.079
Total (1133)	2.0 (1.2)	1.7 (1.0)	2.7 (1.4)	**<0.001**	159	110 (69.2)	49 (30.8)	**<0.001**
Hot drinks								
Broths (9)	3.6 (0.5)	-	3.6 (0.5)	-	-	-	-	-
Coffee (789)	3.6 (1.2)	3.6 (1.2)	4.9 (1.7)	**0.003**	-	-	-	-
Hot chocolate (127)	2.1 (0.9)	-	2.1 (0.9)	-	-	-	-	-
Hot water (1)	1.0 (-)	1.0 (-)	-	-	-	-	-	-
Infusions (48)	2.2 (1.0)	2.2 (1.0)	-	-	-	-	-	-
Milk (64)	3.2 (0.7)	3.2 (0.7)	-	-	-	-	-	-
Total (1038)	3.3 (1.3)	3.5 (1.2)	2.4 (1.2)	**<0.001**	-	-	-	-
Total (3894)	9.1 (10.4)	6.1 (10.4)	12.2 (9.4)	**<0.001**	1511	327 (21.6)	1184 (78.4)	**<0.001**

Abbreviations: SD, standard deviation; PHNQ, products of high nutritional quality; PLNQ products with low quality. Note: ^a^ If the product was located at a height between 80 cm and 170 cm, it was considered to be promoted; ^b^ If a product had been classified as LNQ according to AECOSAN and UK nutrient profiling model criteria, it was considered with LNQ; ^c^ Mann-Whitney U test was used to assess differences between PHNQ and PLNQ, significant *p*-values are highlighted in bold; ^d^ Product promotion was not evaluated for those products that were not in view, because all the product selection panels were located at hand or eye level (*n* = 1677).

## Data Availability

Data are to be made available only via a request to the corresponding author. Data will be provided only after the acceptance and signature of a formal data sharing agreement.

## Supplementary Materials

The following are available online at https://www.mdpi.com/article/10.3390/ijerph18136842/s1, Table S1: Percentages of products classified into the same or opposite category and agreement between the two nutrient profiling models (AECOSAN and UK NPM); Table S2: Percentages of products classified into the same or opposite category and agreement between the two nutrient profiling models (AECOSAN and UK NPM) and the combination of both and processing level classification (NOVA system); Table S3: Simple linear regression analyses examining price by NPMs of products offered on vending machines on campus; Table S4: Binary logistic regression analyses examining promotion by NPMs of products offered on vending machines on campus.
