# Supplementary material for: Availability, Nutritional Profile and Processing Level of Food Products Sold in Vending Machines in a Spanish Public University
Source: Int J Environ Res Public Health. 2021 Jun 25;18(13):6842. doi: 10.3390/ijerph18136842 (PMC8297357; doi:10.3390/ijerph18136842)
Supplement: Supplementary file 1 [file ijerph-18-06842-s001.zip › ijerph-1219516-supplementary.pdf]

Supplemenary materials

Table S1. Percentages of products classified into the same or opposite category and agreement between the two nutrient profiling models (AECOSAN and UK NPM).

	**AECOSAN** [a]				**Kappa coefficient** [b]
	**PLNQ**		**PHNQ**		
	n	**%**	***n***	**%**	
UK NPM[c]					
Cold/hot foods (*n* = 1723)					0.408
PLNQ	1403	81.4	19	1.1	
PHNQ	204	11.8	97	5.6	
Total	1607	93.3	116	6.7	
Cold drinks (*n* = 1133)					0.825
PLNQ	345	30.4	89	7.8	
PHNQ	1	0.1	698	61.6	
Total	346	30.5	787	69.5	
Hot drinks (*n* = 1038)					0.984
PLNQ	146	14.1	4	0.4	
PHNQ	-	-	888	85.5	
Total	146	14.1	892	85.9	
Total (*n* = 3894)					0.837
PLNQ	1894	48.6	112	2.9	
PHNQ	205	5.3	1683	43.2	
Total	2099	53.9	1795	46.1	

Abbreviations: NPM, nutrient profiling model; PHNQ, products of high nutritional quality; PLNQ, products of low nutritional quality. Note: [a] AECOSAN, 2010; [b] The kappa results were interpreted as follows: values ≤0 no agreement, 0.1–0.20 none to slight, 0.21–0.40 fair, 0.41–0.60 moderate, 0.61–0.80 substantial and 0.81–1.00 almost perfect; [c] Department of Health, 2011.

Table S2. Percentages of products classified into the same or opposite category and agreement between the two nutrient profiling models (AECOSAN and UK NPM) and the combination of both and processing level classification (NOVA system).

	NOVA system[a]				Kappa coefficient[b]
	Ultra-processed		Non-ultra-processed		
	n	%	*n*	%	
AECOSAN[c]					
Cold/hot foods (n=1723)					0.047
PLNQ	1607	93.3	-	-	
PHNQ	113	6.7	3	0.2	
Total	1720	99.8	3	0.2	
Cold drinks (n=1133)					0.318
PLNQ	346	30.5	-	-	
PHNQ	446	39.4	341	30.1	
Total	792	69.9	341	30.1	
Hot drinks (n=1038)					0.462
PLNQ	146	14.1	-	-	
PHNQ	220	21.2	672	64.7	
Total	366	35.3	672	64.7	
Total (n=3,894)					0.584
PLNQ	2099	53.9	-	-	
PHNQ	779	20.0	1016	26.1	
Total	2878	73.9	1016	26.1	
UK NPM[d]					
Cold/hot foods (*n* = 1723)					0.016
PLNQ	1422	82.5	-	-	
PHNQ	298	17.3	3	0.2	
Total	1720	99.8	3	0.2	
Cold drinks (*n* = 1133)					0.422
PLNQ	434	38.3	-	-	
PHNQ	358	31.6	341	30.1	
Total	792	69.9	341	30.1	
Hot drinks (*n* = 1038)					0.473
PLNQ	150	14.4	-	-	
PHNQ	216	20.8	672	64.7	
Total	366	35.3	672	64.7	
Total (*n* = 3894)					0.546
PLNQ	2006	51.5	-	-	
PHNQ	872	22.4	1,016	26.1	
Total	2878	73.9	1,016	26.1	
AECOSAN[c]+UK NPM[d]					
Cold/hot foods (n = 1723)					0.015
PLNQ	1607	93.3	-	-	
PHNQ	113	6.6	3	0.2	
Total	1720	99.8	3	0.2	
Cold drinks (*n* = 1133)					0.317
PLNQ	346	30.5	-	-	
PHNQ	446	39.4	341	30.1	
Total	792	69.9	341	30.1	
Hot drinks (*n* = 1038)					0.462
PLNQ	146	14.1	-	-	

PHNQ	220	21.2	672	64.7	
Total	366	35.3	672	64.7	
Total (n = 3894)					0.501
PLNQ	1894	48.6	-	-	
PHNQ	984	25.3	1,016	26.1	
Total	2878	73.9	1,016	26.1	

Abbreviations: NPM, nutrient profiling model; PHNQ, products of high nutritional quality; PLNQ, products of low nutritional quality. Note: [a]Monteiro *et al.*, 2016; [b]The kappa results were interpreted as follows: values ≤0 no agreement, 0.1–0.20 none to slight, 0.21–0.40 fair, 0.41–0.60 moderate, 0.61–0.80 substantial and 0.81–1.00 almost perfect; [c]AECOSAN, 2010; [d]Department of Health of the UK, 2011.

Table S3. Simple linear regression analyses examining price by NPMs of products offered on vending machines on campus.

**Independent variables** [a]	**Price** [b]					
	**Cold/hot foods**		**Cold drinks** [c]		**Hot drinks**	
	**β (95% CI)**	***p*-value**[d]	**β (95% CI)**	***p*-value**[d]	**β (95% CI)**	***p*-value**[d]
AECOSAN criteria	0.93 (0.82, 1.03)	**<0.001**	-0.57 (-0.64, -0.50)	**<0.001**	0.46 (0.39, 0.52)	**<0.001**
UK NPM criteria	0.25 (0.18, 0.32)	**<0.001**	-0.61 (-0.68, -0.54)	**<0.001**	0.45 (0.39, 0.51)	**<0.001**
AECOSAN+UK NPM	0.31 (0.24, 0.39)	**<0.001**	-0.57 (-0.64, -0.50)	**<0.001**	0.46 (0.39, 0.52)	**<0.001**

Abbreviations: NPM, nutrient profiling model. Note: [a] Ref: unfulfillment of the criterion, that is, PLNQ; [b] Log transformation was conducted, as data was not normally distributed; [c] Bottled water (n = 341) was excluded from the analysis; [d] Significant p-values are highlighted in bold.

Table S4. Binary logistic regression analyses examining promotion by NPMs of products offered on vending machines on campus.

Independent variables [a]	Promotion [b]			
	**Cold/hot foods**		**Cold drinks [c,d]**	
	OR (95% CI)	*p*-value [e]	OR (95% CI)	*p*-value [e]
AECOSAN criteria	2.49 (1.36, 4.58)	**0.003**	0.46 (0.30, 0.72)	**0.001**
UK NPM criteria	0.46 (0.35, 0.60)	**<0.001**	0.48 (0.32, 0.71)	**<0.001**
AECOSAN+UK NPM	0.50 (0.38, 0.65)	**<0.001**	0.45 (0.29, 0.70)	**<0.001**

Abbreviations: NPM, nutrient profiling model. Note: [a] Ref: unfulfillment of the criterion, that is, PLNQ; [b] If the product was located at a height between 80 cm and 170 cm, it was considered to be promoted; [c] Bottled water (n = 341) was excluded from the analysis; [d] The products promotion was not evaluated in those products that were not in view; [e] Significant *p*-values are highlighted in bold.
